# Diagnostic Performance of Atherogenic Index of Plasma for Predicting Diabetic Foot Osteomyelitis with Peripheral Artery Disease

**DOI:** 10.3390/jcm13071934

**Published:** 2024-03-27

**Authors:** Sebastián Flores-Escobar, Mateo López-Moral, Marta García-Madrid, Francisco J. Álvaro-Afonso, Aroa Tardáguila-García, José Luis Lázaro-Martínez

**Affiliations:** 1 Diabetic Foot Unit, Clínica Universitaria de Podología, Facultad de Enfermería, Fisioterapia y Podología, Universidad Complutense de Madrid, 28040 Madrid, Spain; jhflores@ucm.es (S.F.-E.); magarc28@ucm.es (M.G.-M.); alvaro@ucm.es (F.J.Á.-A.); aroa.tardaguila@ucm.es (A.T.-G.); diabetes@ucm.es (J.L.L.-M.); 2 Instituto de Investigación Sanitaria del Hospital Clínico San Carlos (IdISSC), 28040 Madrid, Spain

**Keywords:** diabetic foot, diabetic foot infection, diabetic foot osteomyelitis, atherogenic index of plasma

## Abstract

**Background:** This study aims to assess the atherogenic index of plasma (AIP) diagnostic value in detecting diabetic foot osteomyelitis (DFO) among patients with diabetic foot ulcers (DFUs). **Methods:** A prospective cohort study was conducted on 80 patients with DFUs and suspected DFO between January 2022 and December 2023. The primary outcome measures included the diagnosis of DFO, determined by positive microbiological analysis results from bone samples and its correlation with the AIP. Receiver operating characteristic (ROC) curves were utilized to select the optimal diagnostic cut-off points for AIP and post hoc analysis was performed to evaluate the difference in the AIP for diagnosing DFO in patients with and without peripheral arterial disease (PAD). **Results:** The diagnostic potential for DFO in PAD patients of AIP-1 (Log TC/HDL) showed an AUC of 0.914 (*p* < 0.001 [0.832–0.996]), leading to a sensitivity of 83% and a specificity of 85%. By contrast, AIP-2 (Log TG/HDL) demonstrated a slightly lower AUC of 0.841 (*p* < 0.001 [0.716–0.967]), leading to a sensitivity of 76% and a specificity of 74%. **Conclusions:** The AIP tool, with its ideal blend of sensitivity and specificity, aids in predicting DFO effectively. Therefore, clinicians should consider using AIP for patients suffering from PAD and associated DFO.

## 1. Introduction

Diabetic foot ulcers (DFUs) are among the most severe and prevalent complications of diabetes mellitus (DM), affecting approximately 19–34% of people with DM in their lifetime [1]. Roughly half of these ulcers advance to an infection stage [2,3]. Diabetic foot infections (DFI) significantly contribute to morbidity, leading to elevated healthcare costs, diminished physical function, and a lesser quality of life [4,5]. Furthermore, DFIs can extend to the soft tissues or the underlying bone, which results in diabetic foot osteomyelitis (DFO). This condition surfaces in 20% of moderate cases and 50–60% of severe infections, considerably escalating the amputation and mortality rates [6,7,8]. It has been previously reported that DFO can occur in 50–60% of patients with DFI, exhibiting varying levels of severity. Consequently, early and precise diagnosis of DFO is crucial in DFI management, as it helps minimize adverse outcomes [9].

According to the International Working Group on the Diabetic Foot (IWGDF), the best method of diagnosing DFO involves a combination of imaging techniques, such as plain X-ray or Magnetic Resonance Imaging (MRI), a probe-to-bone (PTB) test, and standard laboratory tests. It is important to note, however, that the most reliable test for osteomyelitis is the pathological analysis of the bone sample [5,9,10]. In terms of laboratory tests, the diagnosis of DFO can be informed by an increase in certain inflammatory serum biomarkers. These include white blood cell count (WBC), erythrocyte sedimentation rate (ESR), C-reactive protein (CRP), and procalcitonin (PCT) [5,11,12]. These biomarkers have the advantage of being inexpensive, widely available, and easy to measure. Their main drawback is that they differ depending on the infection’s severity and are unable to definitively confirm or rule out DFO on their own [5,13,14].

The atherogenic index of plasma (AIP) is an alternative to traditional inflammatory biomarkers. It is defined as the logarithmic ratio between triglycerides (TG) or total cholesterol (TC) and high-density lipoprotein-cholesterol (HDL-C) concentrations [15,16]. Prior research suggests a positive correlation between AIP and the risk of cardiovascular diseases and atherosclerosis. Interestingly, during acute inflammation, an inverse correlation has been noted between HDL-C and CRP [17,18,19,20].

So far, only a single study has shown a substantial relationship between the AIP and DFO diagnosis [21]. Consequently, this index’s diagnostic value in identifying osteomyelitis remains ambiguous, warranting further research to substantiate its utility. Therefore, this study seeks to assess the AIP’s diagnostic value in detecting osteomyelitis among patients with DFUs.

## 2. Materials and Methods

### 2.1. Study Design and Participants

This prospective cohort study was conducted at the Diabetic Foot Unit of Complutense, University of Madrid, from January 2022 to December 2023. We studied 80 subjects with DFUs and suspected DFO. The study followed the Standards for Reporting of Diagnostic Accuracy Studies (STARD) 2015 guidelines for diagnostic accuracy studies [22].

We included patients above 18 years old who had been diagnosed with either Type 1 or Type 2 diabetes and who registered an HbA1c of ≤85.8 mmol/mol or <10% within 30 days of the study’s commencement. Other inclusion criteria were the clinical suspicion of DFO in patients with DFUs, surgical treatment of DFUs, and DFUs categorized as stages IIIA, IIIB, IIIC, or IIID as per the University of Texas Diabetic Wound Classification [23], and as grade 3 “O” DFUs according to the PEDIS Classification [24]. Excluded were patients diagnosed with critical limb ischemia and/or previous arterial reconstruction irrespective of the approach (endovascular or bypass) [25], those with acute Charcot foot, and pregnant or lactating women. Patients with insufficient samples for microbiological lab tests were also excluded.

The study received approval from the Ethics Committee at Hospital Clínico San Carlos, Madrid, Spain (C.P.-C.I. 21/762-EC). All participating patients gave their written informed consent prior to participation. In addition, the authors affirmed adherence to the ethical principles of the Declaration of Helsinki [26].

### 2.2. Clinical Evaluation

At the beginning of the study, we gathered demographic details and the clinical characteristics of enrolled patients. These included issues like hypertension, hypercholesterolemia, and smoking history. We also pulled important details from the patient’s medical history like renal, cardiovascular, and retinopathy statuses and recorded details on ulcer location, history of previous ulcers, amputations, and DFO. We noted data related to each patient’s type and average duration of diabetes, as well as the most recent results from blood tests that measured indicators such as glucose, glycated hemoglobin, WBC, neutrophils, triglycerides (TG), TC, high-density lipoprotein cholesterol (HDL-C), low-density lipoprotein cholesterol (LDL-C), albumin, and hemoglobin levels.

Vascular screening was conducted based on various assessments, including distal pulse palpation (dorsalis pedis and posterior tibial pulse), ankle-brachial index (ABI), toe-brachial index (TBI), and transcutaneous oxygen pressure (TcPO_2_). Brachial and ankle systolic blood pressure was measured using an 8-MHz manual Doppler (Huntleigh Healthcare Ltd., Cardiff, UK). Toe systolic blood pressure was measured via digital plethysmography (Systoe, Atys Medical, Madrid, Spain), and TcPO_2_ was analyzed using the TCM4 measuring device (Radiometer, Copenhagen, Denmark) [25]. A diagnosis of peripheral artery disease (PAD) was confirmed in the event a patient exhibited absent distal pulses, an ABI less than 0.9, a TBI less than 0.7 in patients with an ABI greater than 1.4 (indicating arterial calcification), and a TcPO_2_ less than 30 mmHg [27,28]. A neurological assessment was carried out using a Horwell biothesiometer (METEDA S.r.l., San Benedetto del Tronto, Italy) and a Semmes–Weinstein 5.07/10 g monofilament (Novalab Iberica, Madrid, Spain). A diagnosis of diabetic neuropathy was made if patients failed one of these two tests [29].

### 2.3. Clinical Diagnosis of DFO

A combination of PTB tests and plain X-ray scans were used to establish the clinical suspicion of DFO, displaying a high sensitivity and specificity in DFO diagnoses [30]. A PTB test, conducted using sterile Halsted mosquito forceps, was deemed positive if the examiner encountered a gritty or hard surface. For radiographs, a couple of standard views were taken; a positive osteomyelitis diagnosis was made if cortical disruption, periosteal reaction or elevation, bone marrow involvement, or bone sequestrum (devitalized bone distinguishable from healthy bone due to its radio-dense appearance) was visible [31]. If both tests yielded positive results, patients with suspected DFO would be recommended for surgical treatment [32].

The surgeon, a specialist in diabetic foot surgery with over two decades of experience, performed all surgeries using a conservative approach. This method involves the removal of infected bone and non-viable soft tissue, excluding any foot amputation [33]. Bone samples were collected from each patient and analyzed microbiologically to confirm DFO diagnosis.

### 2.4. Microbiological Analysis of Bone Samples

The same surgeon obtained bone samples using an aseptic technique [24]. DFU debridement was performed, followed by the cleansing of the adjacent skin using povidone-iodine and a generous saline rinse to prevent contamination [34]. The authors followed the microbiology laboratory’s protocol for collecting and transporting bone samples [35].

The same microbiologist mechanically homogenized the bone samples in 1 mL of sterile phosphate-buffered saline (PBS, Sigma Aldrich, St Louis, MO, USA) with a pH of 7.4 for 5 min. The homogeneous samples were then cultured and incubated at 35 °C for 48 h. We identified the isolated microorganisms using traditional Gram staining methods, followed by biochemical techniques. These were based on either the API bioMérieux (Marcy, L’Etoile, France) or BBL Crystal ID systems (BD) [6]. Eventually, we classified the results of the bone cultures into two categories: monomicrobial or polymicrobial [36].

### 2.5. Outcome Measures

The primary outcome measures included the diagnosis of DFO, determined by positive microbiological analysis results from bone samples and its correlation with the AIP. Additional long-term outcomes assessed included healing, defined as the ulcer’s complete epithelialization with unbroken skin within 2 weeks, re-amputation (a subsequent amputation performed at the initial site), and major amputation—any cut made above the ankle [37].

### 2.6. Statistical Analysis

We used SPSS version 25.0 (IBM Corp, Armonk, NY, USA) for all statistical analyses. Qualitative variables are demonstrated through frequency distributions and percentages, while quantitative variables are shown as mean and standard deviation. The Shapiro–Wilk test verified the normality of all continuous variables. We reported variables with normal distributions (Shapiro–Wilk test with *p* ≥ 0.05) as mean and standard deviations (SD); meanwhile, those with non-normal distributions (Shapiro–Wilk test with *p* < 0.05) were reported as medians and interquartile ranges. The chi-square and Student’s *t*-tests calculated the differences in clinical attributes of patients with and without DFO for categorical and quantitative variables, respectively. The strength of difference in the effect size was calculated by the Phi coefficient for chi-square test and r coefficient for non-parametric test considering the values >0.01 as a small effect, >0.30 as a medium effect, and >0.50 as a large effect. Cohen’s d was calculated as the effect size for parametric test and the values >0.2, >0.5, and >0.8 were considered as small, moderate, and large effects, respectively. Post hoc analysis evaluated the difference in the AIP in patients with and without PAD towards diagnosing DFO. To select optimal diagnostic cut-off points for the AIP, we utilized receiver operating characteristic (ROC) curves. This index’s sensitivity (S) and specificity (SP) were also calculated. We took *p*-values < 0.05 as significant, considering a 95% confidence interval.

The sampling was based on a nonprobability or convenience method. No existing reference in the literature was found for sample size calculation, as this study is the first to evaluate this particular condition using AIP tool.

## 3. Results

In this study, 80 patients who had either Type 1 or Type 2 diabetes, along with an active DFU and suspected DFO, were examined. Table 1 showcases their demographic features and diabetic complications. Post bone culture examination, DFO was diagnosed in 65% of the patients (52 out of 80). Upon comparison of data, no significant differences were noted between the two groups (with or without DFO) in relation to diabetic complications and past foot issues. The location and properties of the ulcers also did not exhibit any differences between the two sets of patients.

Additionally, we did not find any differences in analytic blood test characteristics between the groups. The blood test results are presented in Table 2.

When analyzing long-term outcomes divided by the presence of DFO, we did not observe any differences between healing rate, re-amputation, and major amputations (Table 3).

### Diagnostic Performance of AIP for Detection of DFO and Post Hoc Analyses

The AIP, a logarithmic transformation of TG/HDL-C and TC/HDL-C ratios, varied between 0.50 ± 0.12 for TC/HDL-C and 0.24 ± 0.13 for TG/HDL-C among the 80 participants. For the 56 patients with osteomyelitis, the mean AIP-1 was 0.53 ± 0.12, and AIP-2 was 0.26 ± 0.15. These values did not differ significantly from the mean AIP-1 and AIP-2 of the control group (*p* = 0.06 and *p* = 0.064, respectively) (Table 2).

In the post hoc analyses, we found statistically significant differences in the presence of PAD (n = 49, 61.3%) between both AIP-1 (0.62 ± 0.1) and AIP-2 (0.35 ± 0.12) when DFO was present, as well as AIP-1 (0.48 ± 0.1) and AIP-2 (0.21 ± 0.1) when DFO was absent; *p* < 0.001 for both AIP-1 and AIP-2.

**Figure 1:**
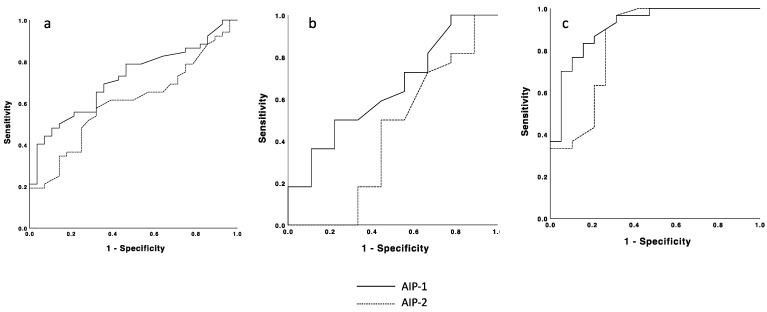
Receiver operating characteristic (ROC) curves for the atherogenic index of plasma (AIP) in the prediction of diabetic foot osteomyelitis. AIP-1, atherogenic index of plasma (Log TC/HDL); AIP-2, atherogenic index of plasma (Log TG/HDL); (**a**) ROC curves for all the samples (N = 80). (**b**) ROC curves for non-peripheral artery disease patients (n = 31). (**c**) ROC curves for peripheral artery disease patients (n = 49).

Table 4 presents the performance characteristics of AIP in patients with DFO stratified for all the sample and PAD patients. The primary outcome measure indicates that both TC/HDL and TG/HDL have a limited capacity to diagnose DFO, as evidenced by the area under the curve (AUC) for TC/HDL at 0.480 (*p* = 0.077, 95% CI [0.342–0.618]) and for TG/HDL at an AUC of 0.527 (*p* = 0.07, 95% CI [0.388–0.666]) (Figure 1). However, when considering only patients with PAD, the diagnostic potential for DFO increases significantly when using AIP-1; this showed an AUC of 0.914 (*p* < 0.001 [0.832–0.996]), leading to a sensitivity of 83% and a specificity of 85%. By contrast, AIP-2 demonstrated a slightly lower AUC of 0.841 (*p* < 0.001 [0.716–0.967]), leading to a sensitivity of 76% and a specificity of 74% (Table 4).

## 4. Discussion

In this prospective study, we examined the relation between AIP values and the presence of DFO coexisting with peripheral artery disease.

An early and precise diagnosis of DFO is pivotal for prompt and proper medical management. It is also critical for preventing systemic sepsis, infection, and possible minor or major amputations. Our findings, however, suggest that the AIP is not a reliable blood test for diagnosing DFO in the general population, irrespective of the logarithmic calculation or the usage of triglycerides (TG) or TC in the formula.

Lower extremity atherosclerosis is speculated to play a central role in DFO development [38]. We noticed a greater diagnostic capability of AIP in patients with PAD compared to non-ischemic patients. Previous literature suggested a potentially strong predictive value for AIP in osteomyelitis cases [21]. This assertion was bolstered by the observed higher prevalence of elevated AIP values in patients with DFUs compared to those without, with even higher AIP values in infected DFU patients versus non-infected ones [21].

Our results demonstrate a similar sensitivity of 83% and a specificity of 85% in the PAD patient cohort to those found by Nie et al. In this study, we assessed both TC/HDL and TG/HDL ratios, as prior research proposed their combined use as a cost-effective and simple blood test for evaluating cardiovascular disease [15,16]. Nonetheless, we found a notable difference in the predictive power of AIP evaluated as a TG/HDL ratio for detecting DFO compared to TC/HDL.

Our research indicates that AIP (TC/HDL) may be a promising tool for suspecting a DFO event. However, other blood tests with more substantiated evidence in the literature, such as CRP, ESR, or procalcitonin (PCT), can also be used [5]. Further analyses should be considered to compare the diagnostic performance of AIP with CRP, ESR, or PCT.

Our findings should be interpreted cautiously due to several study limitations. Firstly, the small sample size, particularly for non-peripheral artery disease patients, may limit our results. Secondly, while we compared AIP with bone culture, following IWGDF guidance, the gold standard for diagnosing DFO is considered to be bone biopsy [39]. Further research should analyze the AIP tool in a cohort of patients with critical limb ischemia, increasing the generalizability of our results; additionally, the study design is noncomparative without a control group to compare the outcomes. Future investigations must be focused on randomized clinical trial designs.

The principal strength of our study, to our awareness, is that it is the inaugural prospective study validating the use of AIP as a novel diagnostic tool for DFO in patients with peripheral artery disease. Additionally, our research was based on certain clinical suspicion according to the IWGDF guidance as the combination of a positive PTB test and the presence of radiological signs consistent with DFO; Nie X et al. [21] resulted in a higher heterogeneity in the suspicion of DFO which can be a limitation of the research.

This study equips clinicians with crucial insights, promoting earlier detection of DFO using AIP. As a result, it could lessen the severity of foot infections, diminish the likelihood of additional amputations, and decrease related costs.

## 5. Conclusions

The AIP tool, with its ideal blend of sensitivity and specificity, aids in diagnosing DFO effectively. Therefore, clinicians should consider using AIP for patients suffering from PAD and associated DFI.

## Figures and Tables

**Table 1:** Patients’ baseline characteristics and diabetes complications (N = 80).

Baseline Characteristics		Patients (N = 80)	Osteomyelitis Patients (n = 52)	Non-Osteomyelitis Patients (n = 28)	*p*-Value 95% CI	Effect Size
Male, n (%)		64 (80%)	41 (78.8%)	23 (82.1%)	0.725	0.039 ^a^
Female, n (%)		16 (20%)	11 (21.2%)	5 (17.9%)		
Type 2 Diabetes, n (%)		74 (92.5%)	49 (94.2%)	25 (89.3%)	0.423	0.09 ^a^
Type 1 Diabetes, n (%)		6 (7.5%)	3 (5.8%)	3 (10.7%)		
Retinopathy, n (%)		25 (31.3%)	17 (32.7%)	8 (28.6%)	0.704	0.042 ^a^
Renal disease, n (%)		17 (21.3%)	13 (25%)	4 (14.3%)	0.264	0.125 ^a^
Hypertension, n (%)		69 (86.3%)	46 (88.5%)	23 (82.1%)	0.434	0.088 ^a^
Hypercholesterolemia, n (%)		57 (71.3%)	39 (75%)	18 (64.3%)	0.313	0.133 ^a^
Cardiovascular disease, n (%)		33 (41.3%)	21 (40.4%)	12 (42.9%)	0.830	0.024 ^a^
Smoking status	Never, n (%)	31(38.8%)	18 (34.6%)	13 (46.4%)	0.244	0.193 ^a^
	Current, n (%)	17 (21.3%)	14 (26.9%)	3 (10.7%)		
	Former, n (%)	32 (40%)	20 (38.5%)	12 (42.9%)		
Ulcer location	Hallux, n (%)	9 (11.3%)	5 (9.6%)	4 (14.3%)	0.468	0.261 ^a^
	1st MTH, n (%)	12 (15%)	9 (17.3%)	3 (10.7%)		
	Central MTH, n (%)	15 (18.8%)	9 (17.3%)	6 (21.4%)		
	Fifth MTH, n (%)	9 (11.3%)	7 (13.5%)	2 (7.1%)		
	Toes, n (%)	27 (33.8%)	18 (34.6%)	9 (32.1%)		
	Midfoot, n (%)	3 (3.8%)	1 (1.9%)	2 (7.1%)		
	Heel, n (%)	2 (2.5%)	2 (3.8%)	-		
	TMA, n (%)	3 (3.8%)	1 (1.9%)	2 (7.1%)		
Previous Ulceration, n (%)		75 (93.8%)	48 (92.3%)	27 (96.4%)	0.468	0.081 ^a^
Previous Amputation, n (%)		46 (57.5%)	29 (55.8%)	15 (53.6%)	0.670	0.048 ^a^
Previous DFO, n (%)		47 (58.8%)	32 (61.5%)	17 (60.7%)	0.490	0.077 ^a^
Body Mass Index, mean ± SD		27.9 ± 6.41	28.1 ± 7.3	27.4 ± 4.4	0.630	0.113 ^b^
Mean age ± SD (years)		70.3 ± 11.4	70.3 ± 10.2	70.2 ± 12.6	0.977	0.007 ^b^
Diabetes mellitus (years), mean ± SD		20.6 ± 11.2	19.2 ± 10.1	23.2 ± 12.8	0.127	0.362 ^b^
Positive probe-to-bone test, n (%)		80 (100%)	52 (100%)	28 (100%)	-	0.153 ^a^
Presence of dorsalis pedis pulse, n (%)		35 (43.8%)	26 (50%)	9 (32.1%)	0.125	0.172 ^a^
Presence of posterior tibial pulse, n (%)		30 (37.5%)	23 (44.2%)	7 (25%)	0.090	0.189 ^a^
Ankle-Brachial Pressure Index, mean ± SD		1.1 ± 0.3	1.1 ± 0.3	1.02 ± 0.3	0.318	0.251 ^b^
Toe-Brachial Pressure Index, mean ± SD		0.67 ± 0.3	0.67 ± 0.26	0.69 ± 0.36	0.788	0.077 ^b^
TcpO_2_ (mmHg), mean ± SD		35.9 ± 12.6	36.5 ± 11.6	35.1 ± 14.4	0.647	0.112 ^b^

SD, standard deviation; CI, confidence interval. ^a^ For categorical variables, chi-square test was used. As the Phi coefficient, 0.01 represents a small effect, 0.30 represents a medium effect, and 0.50 represents a large effect. ^b^ For normally distributed variables, Student’s *t*-test was used for independent samples; effect size was given by Cohen’s d: >0.2 for a small effect, >0.5 for a moderate effect, and >0.8 for a large effect.

**Table 2:** Patients’ analytic blood test results (N = 80).

Analytic Results	Patients (N = 80)	Osteomyelitis Patients (n = 52)	Non-Osteomyelitis Patients (n = 28)	*p*-Value 95% CI	Effect Size
Glycated hemoglobin (%), mean ± SD	7.6 ± 1.3	7.6 ± 1.4	7.54 ± 1.1	0.823	0.005 ^b^
White blood cell count (10^9^/L), mean ± SD	8.2 ± 2.8	8.2 ± 3.1	8.16 ± 2.1	0.932	0.02 ^b^
Hemoglobin (g/dL), mean ± SD	13.4 ± 1.9	13.2 ± 2.03	13.9 ± 1.5	0.117	0.377 ^b^
Neutrophils (10^9^/L), mean ± SD	4.9 ± 1.9	4.95 ± 2.08	4.7 ± 1.8	0.657	0.108 ^b^
Albumin (g/dL), mean ± SD	3.9 ± 0.4	3.99 ± 0.5	3.8 ± 0.3	0.299	0.367 ^b^
TC (mmol/L), mean ± SD	3.81 ± 1.02	3.73 ± 1.01	3.94 ± 1.05	0.403	0.20 ^b^
TG (mmol/L), mean ± SD	1.61 ± 0.82	1.65 ± 0.88	1.54 ± 0.73	0.586	0.132 ^b^
HDL-C (mmol/L), mean ± SD	1.05 ± 0.35	1.06 ± 0.38	1.05 ± 0.20	0.915	0.026 ^b^
LDL-C (mmol/L), mean ± SD	1.99 ± 0.76	1.96 ± 0.78	2.04 ± 0.72	0.696	0.096 ^b^
Glucose (mg/dL), mean ± SD	161.3 ± 57.6	163.4 ± 58.9	157.3 ± 56	0.654	0.105 ^b^
Log TC/HDL (AIP-1), mean ± SD	0.50 ± 0.12	0.53 ± 0.12	0.44 ± 0.1	0.060	0.764 ^b^
Log TG/HDL (AIP-2), mean ± SD	0.24 ± 0.13	0.26 ± 0.15	0.20 ± 0.08	0.064	0.443 ^b^

SD, standard deviation; CI, confidence interval. ^b^ For normally distributed variables, Student’s *t*-test was used for independent samples; effect size was given by Cohen’s d: >0.2 for a small effect, >0.5 for a moderate effect, and >0.8 for a large effect.

**Table 3:** Patient’s long-term outcomes are divided by the presence of DFO (N = 80).

Outcomes	Patients (N = 80)	Osteomyelitis Patients (n = 52)	Non-Osteomyelitis Patients (n = 28)	*p*-Value 95% CI	Effect Size
Healing, mean ± SD	72 (90%)	48 (92.3%)	24 (85.7%)	0.348	0.105 ^a^
Re-amputation, mean ± SD	19 (23.8%)	14 (26.9%)	5 (17.9%)	0.363	0.102 ^a^
Major amputation, mean ± SD	4 (5%)	4 (7.7%)	0 (0%)	0.132	0.168 ^a^

SD, standard deviation; CI, confidence interval. ^a^ For categorical variables, chi-square test was used. As the Phi coefficient, 0.01 represents a small effect, 0.30 represents a medium effect, and 0.50 represents a large effect.

**Table 4:** Performance characteristics of AIP in patients with DFO.

Pooled	All the Sample (N= 80)		Non-PAD Patients (n = 31)		PAD Patients (n = 49)	
	AIP-1	AIP-2	AIP-1	AIP-2	AIP-1	AIP-2
Cut-off point	0.55	0.18	0.55	0.18.	0.55	0.18
AUC (*p*-value [95% CI])	0.717 (0.001 * [0.605–0.828])	0.603 (0.121 [0.479–0.728])	0.646 (0.207 [0.433–0.860])	0.429 (0.542 [0.171–0.687])	0.914 (<0.001 * [0.832–0.996])	0.841 (<0.001 * [0.716–0.967])
Sensitivity	61%	61%	59%	50%	83%	76%
Specificity	68%	60%	66%	66%	85%	74%

AIP-1, atherogenic index of plasma (Log TC/HDL); AIP-2, atherogenic index of plasma (Log TG/HDL). * *p* < 0.05 indicates statistical significance; PAD (Peripheral arterial disease).

## Data Availability

No new data were created or analyzed in this study. Data sharing is not applicable to this article.

## References

[1] Apelqvist J., Bakker K., van Houtum W.H., Schaper N.C. (2008). Practical guidelines on the management and prevention of the diabetic foot. Diabetes Metab. Res. Rev..

[2] Armstrong D.G., Boulton A.J.M., Bus S.A. (2017). Diabetic Foot Ulcers and Their Recurrence. N. Engl. J. Med..

[3] Petersen B.J., Linde-Zwirble W.T., Tan T.W., Rothenberg G.M., Salgado S.J., Bloom J.D., Armstrong D.G. (2022). Higher rates of all-cause mortality and resource utilization during episodes-of-care for diabetic foot ulceration. Diabetes Res. Clin. Pract..

[4] American Diabetes Association (2023). 12. Retinopathy, neuropathy, and foot care: Standards of Care in Diabetes—2023. Diabetes Care.

[5] Senneville É., Albalawi Z., van Asten S.A., Abbas Z.G., Allison G., Aragón-Sánchez J., Embil J.M., Lavery L.A., Alhasan M., Oz O. (2023). IWGDF/IDSA Guidelines on the diagnosis and treatment of diabetes-related foot infections (IWGDF/IDSA 2023). Diabetes Metab. Res. Rev..

[6] Lipsky B.A., Berendt A.R., Cornia P.B., Pile J.C., Peters E.J.G., Armstrong D.G., Deery H.G., Embil J.M., Joseph W.S., Karchmer A.W. (2012). 2012 Infectious Diseases Society of America Clinical Practice Guideline for the Diagnosis and Treatment of Diabetic Foot Infections. Clin. Infect. Dis..

[7] Lázaro-Martínez J.L., Tardáguila-García A., García-Klepzig J.L. (2017). Diagnostic and therapeutic update on diabetic foot osteomyelitis. Endocrinol. Diabetes Nutr..

[8] Caruso P., Maiorino M.I., Scappaticcio L., Porcellini C., Matrone R., Cirillo P., Macera M., Gicchino M., Vietri M.T., Bellastella G. (2023). Biochemical predictors of diabetic foot osteomyelitis: A potential diagnostic role for parathormone. Diabetes Metab. Res. Rev..

[9] Van Asten S.A.V., Nichols A., La Fontaine J., Bhavan K., Peters E.J.G., Lavery L.A. (2017). The value of inflammatory markers to diagnose and monitor diabetic foot osteomyelitis. Int. Wound J..

[10] Álvaro-Afonso F.J., Lázaro-Martínez J.L., Aragón-Sánchez J., García-Morales E., García-Álvarez Y., Molines-Barroso R.J. (2014). Inter-observer reproducibility of diagnosis of diabetic foot osteomyelitis based on a combination of probe-to-bone test and simple radiography. Diabetes Res. Clin. Pract..

[11] Sharma H., Sharma S., Krishnan A., Yuan D., Vangaveti V.N., Malabu U.H., Haleagrahara N. (2022). The efficacy of inflammatory markers in diagnosing infected diabetic foot ulcers and diabetic foot osteomyelitis: Systematic review and meta-analysis. PLoS ONE.

[12] Soleimani Z., Amighi F., Vakili Z., Momen-Heravi M., Moravveji S.A. (2021). Diagnostic value of procalcitonin, erythrocyte sedimentation rate (ESR), quantitative C-reactive protein (CRP) and clinical findings associated with osteomyelitis in patients with diabetic foot. Hum. Antibodies.

[13] Coye T.L., Suludere M.A., Kang G.E., Crisologo P.A., Malone M., Rogers L.C., Lavery L.A. (2023). The infected diabetes-related foot: Comparison of erythrocyte sedementation rate/albumin and C-reactive protein/albumin ratios with erythrocyte sedimentation rate and C-reactive protein to differentiate bone and soft tissue infections. Wound Repair. Regen..

[14] Lavery L.A., Ahn J., Ryan E.C., Bhavan K., Oz O.K., La Fontaine J., Wukich D. (2019). What are the Optimal Cutoff Values for ESR and CRP to Diagnose Osteomyelitis in Patients with Diabetes-related Foot Infections?. Clin. Orthop. Relat. Res..

[15] Dobiášová M., Frohlich J. (2001). The plasma parameter log (TG/HDL-C) as an atherogenic index: Correlation with lipoprotein particle size and esterification rate in apoB-lipoprotein-depleted plasma (FER HDL). Clin. Biochem..

[16] Li Y., Feng Y., Li S., Ma Y., Lin J., Wan J., Zhao M. (2023). The atherogenic index of plasma (AIP) is a predictor for the severity of coronary artery disease. Front. Cardiovasc. Med..

[17] Edwards M.K., Blaha M.J., Loprinzi P.D. (2017). Atherogenic Index of Plasma and Triglyceride/High-Density Lipoprotein Cholesterol Ratio Predict Mortality Risk Better Than Individual Cholesterol Risk Factors, Among an Older Adult Population. Mayo Clin. Proc..

[18] Liu H., Liu K., Pei L., Li S., Zhao J., Zhang K., Zong C., Zhao L., Fang H., Wu J. (2021). Atherogenic Index of Plasma Predicts Outcomes in Acute Ischemic Stroke. Front. Neurol..

[19] Onat A., Can G., Kaya H., Hergenç G. (2010). “Atherogenic index of plasma” (log10 triglyceride/high-density lipoprotein-cholesterol) predicts high blood pressure, diabetes, and vascular events. J. Clin. Lipidol..

[20] Kobayashi J., Tateishi S., Maruyama T., Kudoh A., Murano S. (2003). Marked reduction in serum high-density lipoprotein cholesterol concentrations in a woman with acute inflammation due to diabetic gangrene. Clin. Chim. Acta.

[21] Nie X., Gao L., Wang L., Wang J. (2020). Atherogenic Index of Plasma: A Potential Biomarker for Clinical Diagnosis of Diabetic Foot Osteomyelitis. Surg. Infect..

[22] Cohen J.F., Korevaar D.A., Altman D.G., Bruns D.E., Gatsonis C.A., Hooft L., Irwig L., Levine D., Reitsma J.B., de Vet H.C.W. (2016). STARD 2015 guidelines for reporting diagnostic accuracy studies: Explanation and elaboration. BMJ Open.

[23] Monteiro-Soares M., Hamilton E.J., Russell D.A., Srisawasdi G., Boyko E.J., Mills J.L., Jeffcoate W., Game F. (2024). Classification of foot ulcers in people with diabetes: A systematic review. Diabetes Metab. Res. Rev..

[24] Lipsky B.A., Senneville É., Abbas Z.G., Aragón-Sánchez J., Diggle M., Embil J.M., Kono S., Lavery L.A., Malone M., van Asten S.A. (2020). Guidelines on the diagnosis and treatment of foot infection in persons with diabetes (IWGDF 2019 update). Diabetes Metab. Res. Rev..

[25] Hinchliffe R.J., Forsythe R.O., Apelqvist J., Boyko E.J., Fitridge R., Hong J.P., Katsanos K., Mills J.L., Nikol S., Reekers J. (2020). Guidelines on diagnosis, prognosis and management of peripheral artery disease in patients with a foot ulcer and diabetes (IWGDF 2019 update). Diabetes Metab. Res. Rev..

[26] World Medical Association (2013). World Medical Association Declaration of Helsinki: Ethical Principles for Medical Research Involving Human Subjects. JAMA.

[27] Chuter V., Schaper N., Hinchliffe R., Mills J., Azuma N., Behrendt C.A., Boyko E.J., Conte M.S., Humphries M., Kirksey L. (2024). Performance of non-invasive bedside vascular testing in the prediction of wound healing or amputation among people with foot ulcers in diabetes: A systematic review. Diabetes Metab. Res. Rev..

[28] Norgren L., Hiatt W.R., Dormandy J.A., Nehler M.R., Harris K.A., Fowkes F.G.R. (2007). Inter-Society Consensus for the Management of Peripheral Arterial Disease (TASC II). Eur. J. Vasc. Endovasc. Surg..

[29] Schaper N.C., van Netten J.J., Apelqvist J., Bus S.A., Fitridge R., Game F., Montero-Soares M., Senneveille E., IWGDF Editorial Board (2024). Practical guidelines on the prevention and management of diabetes-related foot disease (IWGDF 2023 update). Diabetes Metab. Res. Rev..

[30] Calvo-Wright M.D.M., Álvaro-Afonso F.J., López-Moral M., García-Álvarez Y., García-Morales E., Lázaro-Martínez J.L. (2023). Is the Combination of Plain X-ray and Probe-to-Bone Test Useful for Diagnosing Diabetic Foot Osteomyelitis? A Systematic Review and Meta-Analysis. J. Clin. Med..

[31] Lavery L.A., Suludere M.A., Ryan E., Crisologo P.A., Tarricone A., Malone M., Oz O.K. (2024). The infected diabetic foot: Analysis of diabetic and non-diabetic foot infections. Wound Repair Regen..

[32] Mutluoglu M., Lipsky B.A. (2017). Non-surgical treatment of diabetic foot osteomyelitis. Lancet Diabetes Endocrinol..

[33] Aragón-Sánchez J. (2010). Treatment of diabetic foot osteomyelitis: A surgical critique. Int. J. Low. Extrem. Wounds.

[34] Tardáguila-García A., Lázaro-Martínez J.L., Sanz-Corbalán I., García-Álvarez Y., Álvaro-Afonso F.J., García-Morales E. (2019). Correlation between Empirical Antibiotic Therapy and Bone Culture Results in Patients with Osteomyelitis. Adv. Ski. Wound Care.

[35] Ramanujam C.L., Han D., Zgonis T. (2018). Medical Imaging and Laboratory Analysis of Diagnostic Accuracy in 107 Consecutive Hospitalized Patients With Diabetic Foot Osteomyelitis and Partial Foot Amputations. Foot Ankle Spec..

[36] Berendt A., Peters K., Embil J., Eneroth M., Hinchliffe R.J., Jeffcoate W.J., Lipsky B.A., Senneville E., The J., Valk G.D. (2008). Diabetic foot osteomyelitis: A progress report on diagnosis and a systematic review of treatment. Diabetes Metab. Res. Rev..

[37] van Netten J.J., Bus S.A., Apelqvist J., Chen P., Chuter V., Fitridge R., Game F., Hinchliffe R.J., Lazzarini P.A., Mills J. (2023). Definitions and criteria for diabetes-related foot disease (IWGDF 2023 update). Diabetes Metab. Res. Rev.

[38] Davidson L. (2017). Don’t forget peripheral arterial disease in diabetic foot osteomyelitis. Can. Med. Assoc. J..

[39] Hassold N., Bihan H., Moumba Y.P., Poilane I., Méchaï F., Assad N., Labbe-Gentils V., Sal M., Koutcha O.N., Martin A. (2024). BedBiopsy: Diagnostic performance of bedside ultrasound-guided bone biopsies for the management of diabetic foot infection. Diabetes Metab..

